# Acknowledgement to Reviewers of *Methods and Protocols* in 2018

**DOI:** 10.3390/mps2010010

**Published:** 2019-01-18

**Authors:** MPs Editorial Office

**Affiliations:** MDPI, St. Alban-Anlage 66, 4052 Basel, Switzerland

Rigorous peer-review is the corner-stone of high-quality academic publishing. The editorial team greatly appreciates the reviewers who contributed their knowledge and expertise to the journal’s editorial process over the past 12 months. In 2018, a total of 46 papers were published in the journal, with a median time to first decision of 23 days and a median time to publication of 46.5 days. The editors would like to express their sincere gratitude to the following reviewers for their cooperation and dedication in 2018:

Abu-Reziq, RaedLiu, HongweiAhonen, MerjaLocatelli, MarcelloAkhremitchev, Boris B.Lou, FangfeiAmor, RumeloLu, ZhongjingAnand, SwatiLukinavicius, GrazvydasAshfaq, Mohammad KhalidMacori, GuerrinoBach, StephanMagarati, MayaBarakate, AbdellahMaisner, AndreaBarber, Paul R.Makarov, AlexanderBarbosa Pereira, CarinaMalmberg, PerBarbu, MagdaManzo, CarloBaum, ChristelMartin, ArnaudBernard, VeroniqueMasago, YoshifumiBertolino, PascalMatise, MichaelBerton, PaulaMatsumoto, TaroBirner-Gruenberger, RuthMattiuzzo, GiadaBisson, JonathanMc Hugh, LouiseBoldogh, IstvanMcLeroy, KennethBosse, Jens B.McNevin, DennisBrameshuber, MarioMenet, Marie ClaudeBruegmann, TobiasMeng, XiangchaoCalvano, Cosima DamianaMetzger, SabineCameron, Simon J. S.Miranda-Castro, RebecaChamakura, KarthikMishra, NigamChaudhuri, ArunimaMohan, Ganesh Babu MalliChauhan, Raj DeepikaMoore, Matthew D.Chen, LingxinMorley, ValerieChen, LongNakamura, AsakoChen, Yu-ChihNiebur, Glen L.Cheng, ZheNikolic, IvaChua, Mei-SzeNilsson, HenrikCinnamon, YuvalNzelu, ChukwunonsoConcolino, DanielaOrtiz, InmaculadaCostello, Ben De LacyOtaki, JojiCrespan, EmmanuelePalacio, EdwinDawson, JonathanPathmasiri, WimalDe Freitas Fraga, Hugo PachecoPeeples, MarkDe Vita, ValerioPégard, NicolasDever, DanielPérez, SoledadDey, SoumyadeepPetersen, BjörnDhakal, SomaPijnappels, DaniëlDobrzynski, PiotrPinto, Carmelo GarcíaEichmeier, AlesPires, Ricardo HugoEnke, RayPrieto Vidal, NataliaFagerquist, Clifton K.Qin, JingFelgueiras, HelenaQuesada, Jose Ignacio PriegoFernández-Soto, PedroQuirino, Joselito P.Ferrante, PatriziaRab, PetrFlieger, JolantaRatto, Gian MicheleFujiwara, KeiRichter, ClaudiaGao, MinRizzoli, Silvio O.Garcia, RicardoRobben, JohanGaudiano, BrandonRocca, StefanoGavrilov, DimitarRoig-Navarro, Antoni FrancescGeerlof, ArieSawatsky, BevanGewurz, Benjamin E.Scaal, MartinGophna, UriSeaman, MichaelGoyal, RajniSengupta, ArjunGregáň, JurajŞenilă, MarinGriga, MiroslavSergeant, KjellHan, YongshengSeth, RataneshHata, AkihikoShao, LeiHatoum-Aslan, AsmaSillerio Quintana, ManuelHeijs, BramSimonds, VanessaHiggins, LorieSommerer, NicolasHilszczańska, DorotaSottile, FrancescoHonda, ShunichiStaines, KatherineHosseini Nejad, ElhamStavrianidi, AndreyHowden, SaraSun, ChenHu, ChenlinTanimori, ShinjiHughes, StevenTanner, NathanJaffe, EileenTemple, LouiseJohnston, Callum MichaelTerrier, OlivierJones, PhilipThies, StephanKale, AbhijitThuzar, MoeKelly, Christopher V.To, Kin-YingKoehler, PeterTurk, RolfKoel, MihkelUlcese, UmbertoKong, DemingUnal, ElcinKovacic, Melinda ButschVashist, Sandeep K.Krasnyanski, SergeiVemula, HarikaKuo, Dong-HauVisscher, Marty O.Kurien, Biji TheyilamannilVon Eckardstein, ArnoldKuruma, YutetsuWu, JinhuKusters, IljaYazdan-Shahmorad, AzadehLai, Chin WeiZeng, ShaoqunLamere, MPA, ACC, MichelleZhao, ZongshanLamperska, WeronikaZheng, ZhenrongLehnherr, HansjörgZhou, HongyiLeitner, AlexanderZhou, ZhixiongLi, ChenyuZineldin, MosadLi, Ka Wan

